# Altered expression of Csnk1a1p in Autism Spectrum Disorder in Iranian population: case-control study

**DOI:** 10.1038/s41598-024-77603-3

**Published:** 2024-11-16

**Authors:** Zahra Rahmani, Dina Rahmani, Marie Saghaeian Jazi, Mohammad-Reza Ghasemi, Hossein Sadeghi, Mohammad Miryounesi, Katayoon Razjouyan, Mohammad Reza Fayyazi Bordbar

**Affiliations:** 1 Psychiatry and Behavioral Sciences Research Center, Mashhad University of Medical Sciences, Mashhad, Iran; 2 Metabolic Disorders Research Center, Golestan University of Medical Sciences, Gorgan, Iran; 3 Department of Medical Genetics, Faculty of Medicine, Shahid Beheshti University of Medical Sciences, Tehran, Iran; 4 Center for Comprehensive Genetic Services, Shahid Beheshti University of Medical Sciences, Tehran, Iran; 5 Genomic Research Center, Shahid Beheshti University of Medical Sciences, Tehran, Iran; 6 Psychiatric department, Shahid Beheshti University of Medical Sciences, Tehran, Iran

**Keywords:** Autism spectrum disorder, DISC2, Linc00945, Foxg1-as1, Csnk1a1p, Evf2, Genetics, Molecular biology, Neuroscience, Biomarkers, Molecular medicine, Risk factors

## Abstract

Over the past decade, substantial scientific evidence has showed that long non-coding RNAs (lncRNAs) are extensively expressed and play a crucial role in gene modulation through a diverse range of transcriptional, and post-transcriptional mechanisms. Recent discoveries have emphasized the involvement of lncRNAs in maintaining cellular homeostasis and neurogenesis in the brain. Accumulating reports identified dysregulated lncRNAs associated with psychiatric disorders, including autism. In this study, we examined the expression levels of DISC2, Linc00945, Foxg1-as1, Csnk1a1p, and Evf2 lncRNAs in blood samples from 21 clinically diagnosed autistic patients based on the Diagnostic and Statistical Manual of Mental Disorders criteria-5th edition (DSM-5), compared to age, sex, and ethnically-matched 25 healthy individuals. RNA extraction and cDNA synthesis were performed, followed by real-time PCR for quantification of lncRNAs expression levels. Receiver operating characteristic (ROC) curve analysis was used to evaluate biomarker potential. Additionally, we investigated the correlation between gene expression levels and autism comorbidities. Our results showed a significant decrease in Csnk1a1p expression in patients with autism spectrum disorder (ASD) compared to healthy children (P value = 0.0008). ROC curve analysis indicated that Csnk1a1p expression levels could effectively discriminate patients from healthy controls (AUC = 0.837, P value = 0.000284). No significant differences were observed between Csnk1a1p expression levels and comorbidity with ADHD or intellectual disability (p-value > 0.05). Based on these findings, Csnk1a1p may play a significant role in autistic patients and could serve as a potential biomarker for diagnostic and predictive purposes, as well as a therapeutic target.

## Introduction

Long non-coding RNAs (lncRNAs), a subclass of non-coding RNAs exceeding 200 nucleotides in length. It has been discovered that numerous lncRNAs contain short open reading frames (sORFs) that encode peptides and small proteins^1^. The Human GENCODE project reports over 19,000 lncRNA genes in the human genome, highlighting their remarkable diversity. Current research suggests that lncRNA biogenesis is closely linked to their cellular fate and function. Although the precise functions of most lncRNAs remain elusive, it is evident that numerous lncRNAs play critical roles in various cellular processes^2,3^. LncRNAs can modulate gene expression through interactions with proteins, lncRNA-protein complexes (lncRNP), and chromatin modifiers. These interactions may alter the binding and activity of target DNA regions in cis or trans, ultimately affecting gene expression. LncRNAs can also act as decoys for specific chromatin modifiers, regulating their availability for interaction with target gene promoters and controlling gene expression^4^. Moreover, lncRNPs play a critical role in preserving genome integrity and function during instances of DNA damage. Several studies have revealed that lncRNAs are engaged in scaffolding, nuclear condensate, cytoplasmic mRNA stability and translation regulation, interfering with signaling pathways, and affecting a wide range of cellular functions^5^. It has been demonstrated that roughly 40% of the detected lncRNAs are specifically transcribed in the brain, displaying evolutionary conservation, and frequently involved in the modulation of protein-coding genes with specific roles in neurogenesis^6^. Advancements in genomics-related research have illuminated the significant roles of lncRNAs in neuronal physiology, like neurotransmitter release, synaptogenesis, and neuronal plasticity. Additionally, lncRNAs have been found to play a vital role in nervous system (NS) development, differentiation, and growth. Consequently, lncRNA dysregulation may have implications for various neurological and psychiatric disorders, including Parkinson’s disease, Huntington’s disease, amyotrophic lateral sclerosis, Alzheimer’s disease, brain tumors, and autism spectrum disorder (ASD)^7^. Autism, a heterogeneous neurodevelopmental disorder with a prevalence rate of one in 54 (1.85%), exhibits a global mean age at diagnosis ranging from 38 to 120 months^8,9^. Despite the lack of reliable biomarkers, autism diagnosis is based on two primary domains: social communication and restricted, repetitive, or unusual sensory-motor behaviors^10^. Risk factors for ASD encompass a multitude of aspects, such as advanced parental age, short inter-pregnancy intervals, certain metabolic conditions or inflammation in the mother, preterm birth, and low birth weight in newborns. Additionally, exposure to air pollutants and maternal stressors during pregnancy have been linked to an elevated risk of ASD^9^.

Research identified a microdeletion in chromosome 16p11.2, as well as maternal chromosomal abnormalities on 15q, along with less common deletions or duplications on chromosomes 2q and 22q, which are associated with ASD risk^11^. Recent studies have revealed the involvement of lncRNAs in autism spectrum disorder, where they contribute to gene expression modulation through transcriptional, post-transcriptional, and epigenetic mechanisms^12–14^. Altered lncRNA levels have been reported in ASD cases, including CCAT1, CCAT2, DISC2, LOC101928237, LRRC2-AS1, and PRKAR2A-AS1^15,16^. In this study, we selected five lncRNAs (DISC2, Linc00945, Foxg1-as1, Csnk1a1p, and Evf2) for quantifying their expression in blood samples of children with ASD compared to healthy control children. This research marks the first time such a study (five lncRNAs expression) has been conducted in Iran.

## Materials and methods

### Patients and specimens

This case-control study included 21 ASD patients and 25 age-, sex-, and ethnicity-matched healthy children, selected using convenience sampling methodology. The Diagnostic and Statistical Manual of Mental Disorders, 5th edition (DSM-5), was used to diagnose patients^17^. Participants were referred to the Medical Genetic Clinic of the Center for Comprehensive Genetic Services (CCGS) at Shahid Beheshti University of Medical Sciences (SBMU) from the psychiatric department of Imam Hossein Hospital in Tehran, Iran, between 2020 and 2021. Normal children were determined to have no neuropsychiatric disorders or developmental delays based on their medical history. Five mL blood samples were collected from both children with ASD and normal children for RNA isolation purposes. Written informed consent was obtained from the parents of all participants, and the study protocol was approved by the ethical committee of Mashhad University of Medical Science (IR.MUMS.MEDICAL.REC.1397.247). All research methods adhered to relevant guidelines and regulations.

## Evaluation of lncRNAs expression in blood specimens

RNA was isolated from blood samples utilizing a commercial kit (GeneAll, Germany), according the manufacturer’s recommended protocol. Subsequently, cDNA synthesis was performed using approximately 2 µg of RNA from each sample, using commercial cDNA synthesis kit from Parstous (IRAN). The expression levels of lncRNAs were then quantified according to the protocol, using RealQ Plus 2x Master Mix (Amplicon, Denmark). Beta-2-Microglobulin (B2M) was used as an internal control. The expression levels of lncRNAs were analyzed using the 2-delta-delta-Ct (2-ddCt) formula. The primer sequences used in this study are presented in Table 1. LncRNA expression assay was conducted at the Genomic Research Center, Shahid Beheshti University of Medical Sciences, Iran, between April and November 2022.

**Table 1:** Details about nucleotide sequence of lncRNA’s and B2M primers and amplified sequences used in the study.

Gene names	Primer names	Nucleotide sequence	Product size (bp)
DISC2	Forward Reverse	GGTGAGGTCTTTAGCCGTCC CTTTGCCCATGCTGTGAATGT	135
Linc00945	Forward Reverse	AGCAGGGAGGTCGGCATC CCTCAGTTTCCTCTATCATAC	130
Foxg1-as1	Forward Reverse	GCATACCATAGAGGAGAGG GGCTGTGAGTAAGAGAGG	164
Csnk1a1p	Forward Reverse	CCCAAACCCCAACAGGGTAT TAAATGCAGAGGGGCTGACC	127
Evf2	Forward Reverse	GGATTCTGTGTGGGGTTGGA CATGGGAGCACTCAGCCTAC	187
B2M	Forward Reverse	TGTCTTTCAGCAAGGACTGGT TGCTTACATGTCTCGATCCCAC	143

### Statistical analysis

LncRNA expression levels were analyzed using GraphPad Prism (V 8.2) and SPSS^®^ 20.0 software (SPSS lnc, Chicago, IL, USA). The Shapiro-Wilk test was employed to assess normal distribution, and subsequently, the Mann-Whitney U test was applied to compare expression levels between cases and controls. Spearman’s rho test was utilized for correlation analysis of gene expression data. Data were expressed as mean ± standard error of mean (SE). P-values < 0.05 were considered statistically significant. To evaluate the diagnostic value, ROC curve was established. Additionally, the Mann-Whitney U test was used to analyze the differences between gene expression level and autism comorbidity with ADHD, while the Kruskal-Wallis test was employed for comparison between Comorbidity with intellectual statement.

## Results

**Fig. 1:**
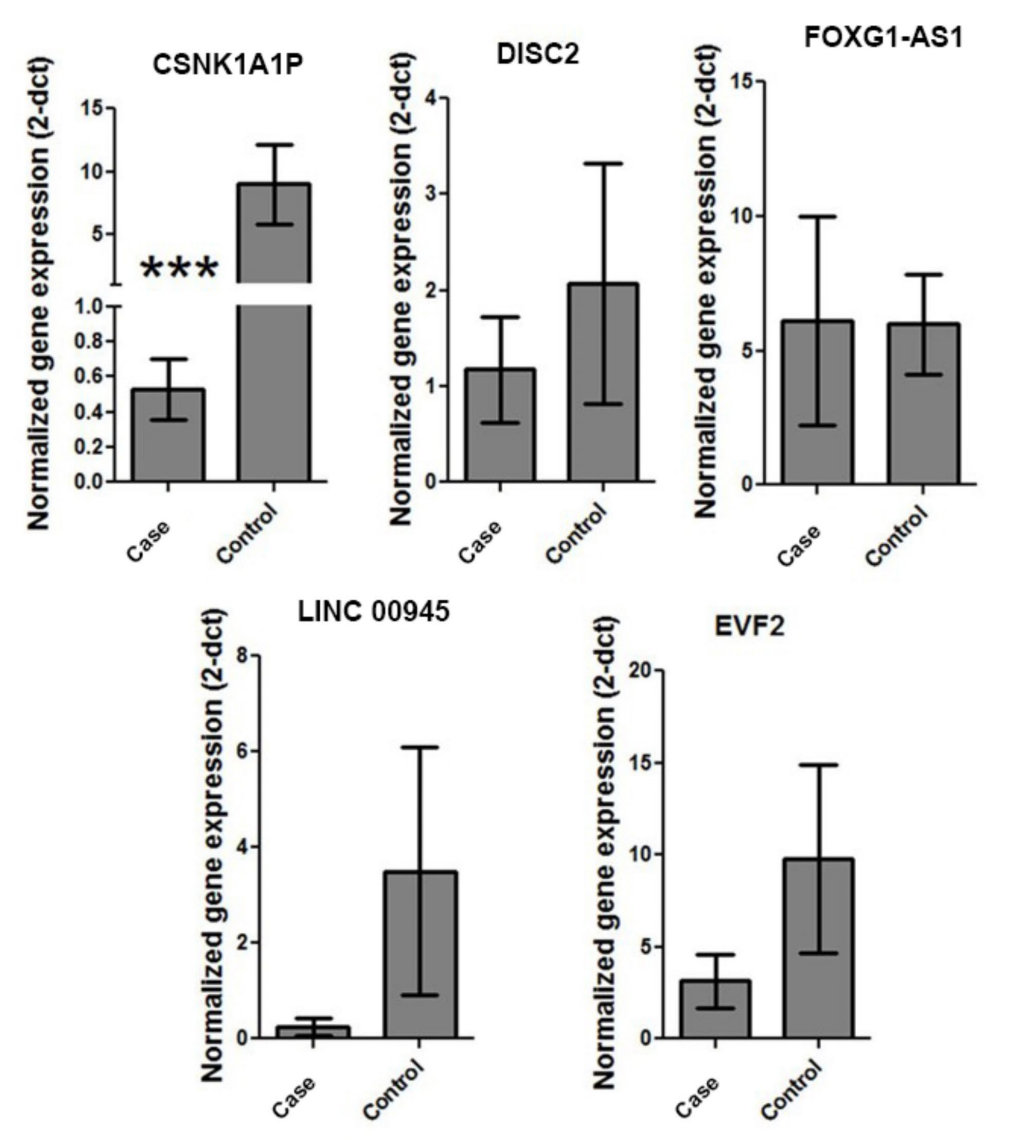
Graphical representation of expression levels of DISC2, Linc00945, Foxg1-as1, Csnk1a1p, and Evf2 in ASD cases and healthy children.

**Fig. 2:**
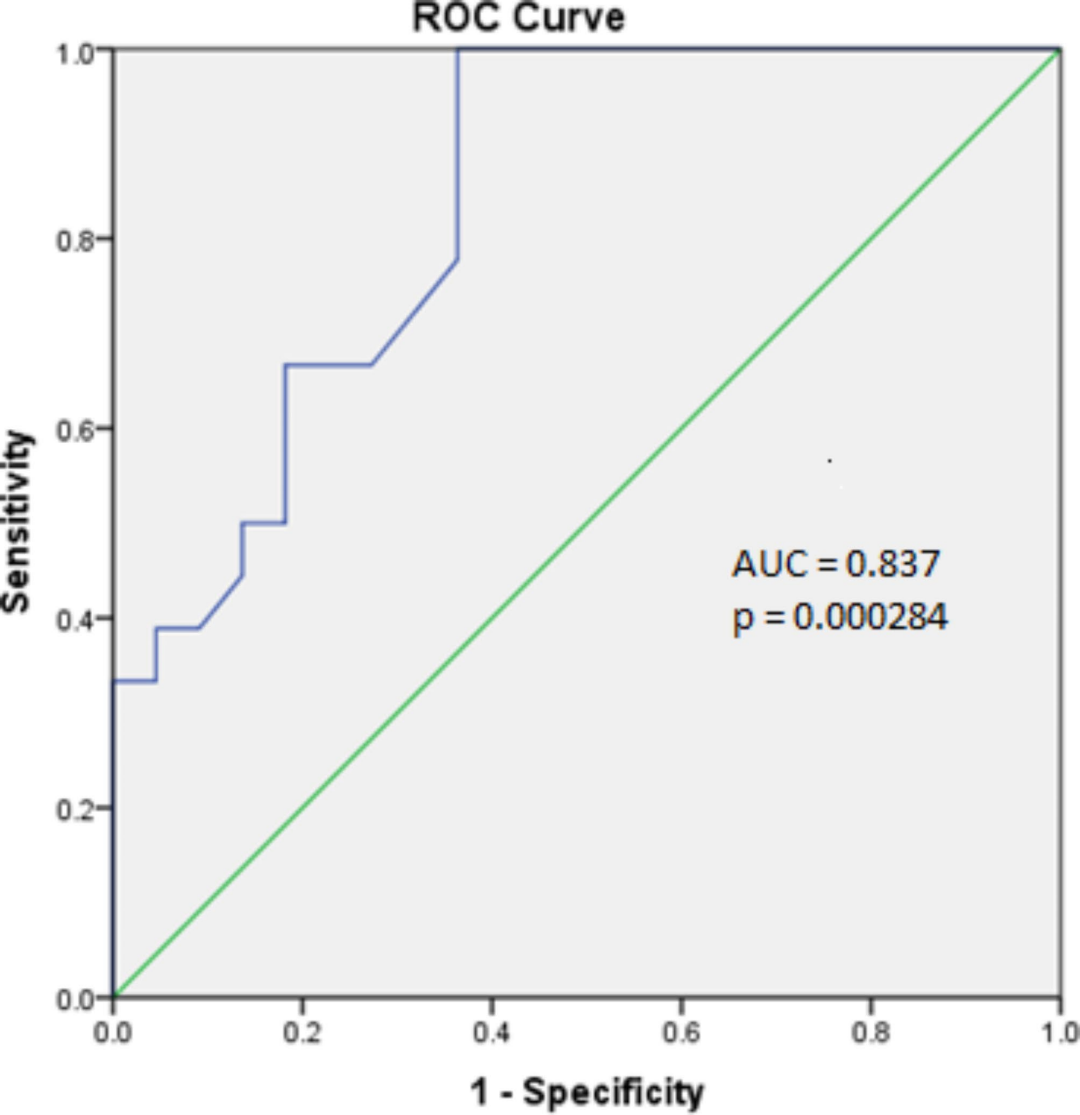
Receiver operating characteristic (ROC) curves. ROC curves were created for Csnk1a1p expression levels in ASD patients versus healthy children.

The study involved comparing 21 patients (15 males and 6 females; male/female ratio: 2.5/1) aged 3 to 17 years old with autism were compared with 25 age-, sex-, and ethnicity-matched controls, aged from 2 to 17 years old, who had no personal history of developmental delays or neuropsychiatric disorders. Additional demographic and clinical details of patients can be found in^18^. The expression levels of DISC2, Linc00945, Foxg1-as1, Csnk1a1p, and Evf2 in the blood of 21 ASD patients and 25 healthy controls were compared. As shown in Fig. 1, the normalized expression level of the Csnk1a1p gene is significantly lower in ASD patients compared to controls (mean ± SE = 0.53 ± 0.17 in ASD vs. 8.9 ± 3.1 in controls; Mann-Whitney test p-value = 0.0008). Additionally, ROC curve indicated a significant diagnostic ability for Csnk1a1p in ASD patients, with the area under the ROC curve (AUC) was 0.837 (lower bound 0.71 to upper bound 0.95) indicating significant discrimination potential (p-value = 0.000284) as a biomarker. At a cut-off value of dCt = 0.4 (positive if ≥ 0.4), this gene expression test for Csnk1a1p yielded a sensitivity of 72% and a specificity of 69% (Fig. 2). No significant difference was observed between the expression levels of Csnk1a1p and comorbidities with ADHD or intellectual conditions (Table 2). Although the analysis revealed no statistically significant difference in the expression levels of other genes (DISC2, Foxg1-as1, Linc00945, and Evf2) in the ASD group compared to controls, a lower mean expression was specially observed for Linc00945 (0.24 ± 0.17 vs. 3.4 ± 2.5, p-value = 0.07) and EVF2 (3.149 ± 1.4 vs. 9.786 ± 5.1, p-value = 0.09) in the ASD group. Moreover, a significant direct correlation was found among the expression levels of all evaluated genes in the studied population, as presented in (Table 3).

**Table 2:** Comparison of the expression levels of Csnk1a1p and comorbidity with ADHD and intellectual statement.

Csnk1a1p		Mean ± (SE)	*P*-value
Comorbidity with intellectual statement	Normal	28.94 ± (0.84)	0.57*
	Mild	29.08 ± (0.36)	
	Moderate	29.32 ± (0.44)	
Comorbidity with ADHD	Yes	29.21 ± (0.33)	0.77**
	No	28.98 ± (0.77)	

*P-value obtained from Kruskal-Wallis test. ** P-value obtained from Mann-Whitney test.

**Table 3:** Expression levels of lncRNAs in children with ASD and controls.

Correlations						
Spearman’s rho		Csnk1a1p	DISC2	Foxg1-as1	Linc00945	Evf2
Csnk1a1p	Correlation Coefficient	1.000	0.558^**^	0.718^**^	0.678^**^	0.499^**^
	Sig. (2-tailed)	.	0.000	0.000	0.000	0.001
DISC2	Correlation Coefficient	0.558^**^	1.000	0.742^**^	0.888^**^	0.715^**^
	Sig. (2-tailed)	0.000	.	0.000	0.000	0.000
Foxg1-as1	Correlation Coefficient	0.718^**^	0.742^**^	1.000	0.699^**^	0.648^**^
	Sig. (2-tailed)	0.000	0.000	.	0.000	0.000
Linc00945	Correlation Coefficient	0.678^**^	0.888^**^	0.699^**^	1.000	0.740^**^
	Sig. (2-tailed)	0.000	0.000	0.000	.	0.000
Evf2	Correlation Coefficient	0.499^**^	0.715^**^	0.648^**^	0.740^**^	1.000
	Sig. (2-tailed)	0.001	0.000	0.000	0.000	.

**. Correlation is significant at the 0.01 level (2-tailed).

## Discussion

In this study, we evaluated the expression levels of five lncRNAs in children with ASD and controls. Our findings revealed a reduced expression level of Csnk1a1p, a processed pseudogene, in ASD cases compared to healthy children. Furthermore, ROC curve analysis confirmed that Csnk1a1p expression levels could effectively discriminate patients from healthy control subjects, indicating its potential utility as an autism biomarker. Processed pseudogenes are copies of messenger RNAs that have undergone reverse transcription into cDNA and have been inserted into the genome via the enzymatic activities of active L1 elements^19^. Numerous studies have showed the involvement of lncRNAs in the development and differentiation of the central nervous system (CNS), including the maintenance of pluripotency, cell fate, neurogenesis, and migration. Specific lncRNAs, such as RMST, TUNA, DALI, and PAUPAR, have been involve in the neurogenic commitment of pluripotent embryonic stem cells^20^. Moreover, evidence suggests that lncRNAs play a role in the regulation of neural genes in non-neural cells and brain tissue patterning^12^. The role of lncRNAs in learning, memory, and cognition can be attributed to their involvement in synaptogenesis and neuronal plasticity. Previous studies have emphasized the critical role of lncRNA dysregulation as a contributing factor in the pathogenesis of various disorders. Gene expression, a quantitative trait with a complex nature, often demonstrates variability in results concerning gene expression levels across different studies. This variation can be attributed to a multitude of factors, such as study design, population variability, sample size, experimental methods, tissue-specific characteristics, and data analysis techniques^21^. A comprehensive understanding of these elements is crucial when comparing and interpreting findings from various research studies. In our investigation, we quantified the expression levels of five lncRNAs in the blood samples of ASD children and controls. Our analysis revealed that none of the lncRNAs DISC2, Linc00945, Foxg1-as1, and Evf2 exhibited a correlation between their expression levels and ASD in comparison with healthy controls. In contrast to previous research suggesting an association between DISC2 and FOXg1-as expression levels and autism in patients compared to healthy controls, our results may differ due to the sample size and study design employed for DISC2 analysis, as well as the utilization of different tissues for FOXg1-as analysis. Notably, Tamizkar et al. observed a significant increase in DISC2 expression in children with ASD^16^), while Williams et al. demonstrated a connection between DISC2 haploinsufficiency and the neurological phenotype of autism^22^. Furthermore, elevated DISC2 lncRNA expression has been reported in bipolar disorder, a condition that co-occurs with ASD^23^. Velmeshev et al. found that Foxg1-as1 expression levels exhibited a non-significant but appreciable difference in postmortem brain samples of individuals with autism compared to control individuals^24^. In the present study, the merely gene identified with differential expression between children with ASD and controls was Csnk1a1p. This gene was found to be downregulated in ASD patients compared to the control group. Located in the 15q14 region, CSNK1A1P1 has been linked to astrocytoma, as reported by^25^. Szikszai et al. reported an association between decreased expression of the processed pseudogene Csnk1a1p and the disruption of transcription and translation processes, ultimately impacting gene expression and protein synthesis. Additionally, reduced Csnk1a1p levels were connected to poly (A) RNA binding^26^. it has been mentioned hat overexpressed Poly(A) binding proteins (PABPs), which play a crucial role in RNA polyadenylation, translation initiation, and mRNA stability, can enhance Long interspersed nuclear elements 1 (L1 elements or LINE1) activity. In other words, the knockdown of the PABPC1 inhibitor PAIP2 gene leads to upregulated L1 retrotransposition. Moreover, L1 elements are believed to moderate the retrotransposition of other protein- coding mRNA and generated processed pseudogenes. Unregulated retrotransposition has been linked to autism^27^. As mentioned earlier, decreased expression of Csnk1a1p1 is associated with disrupted gene expression, which may potentially impact RNA polymerase function in transcription. Recent studies have reported that neurodevelopmental disorders like autism exhibit craniofacial anomalies that may be attributed to the dysregulation of RNA Polymerases I (Pol I) and III (Pol III) function, suggesting a connection between Pol I and Pol III perturbation and these features^28,29^. The findings of this study suggest that Csnk1a1p may play a significant role in patients with ASD, indicating its potential diagnostic or predictive potential as a biomarker and a possible target for modern therapies. Despite limited knowledge about Csnk1a1p lncRNA, identifying its potential target genes and understanding its regulatory mechanisms could greatly contribute to advancing our understanding of ASD. Although limited in sample size due to the pandemic and sampling difficulties, this study provides valuable insights into LncRNAs expression in blood samples of individuals with autism. To strengthen these findings, we suggest future research with larger sample sizes to yield credible results that contribute to a better understanding of lncRNAs expression.

## Conclusions

Our current study presents novel findings demonstrating, for the first time, a significant downregulation of Csnk1a1p in individuals with Autism compared to healthy children which needs to be considered as a novel regulatory mechanism. The observed downregulation of Csnk1a1p may play a critical role in the molecular pathophysiology of ASD, suggesting its potential utility as a diagnostic or prognostic biomarker for autism. Recognizing the importance of these findings, future research should focus on further investigating the functional implications of Csnk1a1p downregulation in ASD. Mechanistic studies should explore the downstream effects of its dysregulation on gene expression and cellular function.

## Data Availability

The datasets during and/or analyzed during the current study are available from the corresponding author on reasonable request.

## Abbreviations

LncRNAs: Long non coding RNAs
LncRNP: LncRNA protein complexes
NS: Nervous system
ASD: Autism spectrum disorder
CNS: Central nervous system
SE: Standard error of mean
